# Antibodies against MYC-Associated Zinc Finger Protein: An Independent Marker in Acute Coronary Syndrome?

**DOI:** 10.3389/fimmu.2017.01595

**Published:** 2017-11-21

**Authors:** Diana Ernst, Christian Widera, Niklas T. Baerlecken, Wolfgang Schlumberger, Cornelia Daehnrich, Reinhold E. Schmidt, Katja Gabrysch, Lars Wallentin, Torsten Witte

**Affiliations:** ^1^Clinic of Rheumatology and Immunology, Hannover Medical School, Hannover, Germany; ^2^Department of Cardiology, Heart Center Oldenburg, European Medical School Oldenburg-Groningen, Carl von Ossietzky University Oldenburg, Oldenburg, Germany; ^3^Euroimmun, Medizinische Labordiagnostika AG, Lübeck, Germany; ^4^Uppsala Clinical Research Center, Uppsala University, Uppsala, Sweden; ^5^Department of Medical Sciences, Cardiology, Uppsala Clinical Research Center, Uppsala University, Uppsala, Sweden

**Keywords:** antibodies, MYC-associated zinc finger protein, acute coronary syndrome, cardiac risk factor, atherosclerosis

## Abstract

**Introduction:**

Atherosclerosis is considered the pathophysiology underlying cardiovascular (CVD), cerebrovascular, and peripheral vascular diseases. Evidence supporting an autoimmune component is emerging, with imaging studies correlating MYC-associated zinc finger protein antibody (MAZ-Ab) optical density (OD) with plaque activity. This study compares MAZ-Ab OD on ELISA testing among patients presenting with acute coronary syndromes (ACSs) to healthy controls and investigates the association of MAZ-Ab to traditional CVD risk factors.

**Methods:**

Patients admitted with ACSs between August 2007 and July 2011 were included. Serum samples taken at presentation were retrospectively tested for MAZ-Ab and compared with serum from healthy volunteers with no CVD risk factors. Large-scale assessment of post-ACS prognostic relevance was performed using the established PLATO cohort.

**Results:**

In total 174 ACS patients and 96 controls were included. Among ACS patients, median MAZ-Ab OD was higher compared with controls (0.46 vs. 0.27; *p* = 0.001). Although the majority of ACS patients (116/174; 67%) had suffered from a ST-elevation myocardial infarction, no significant differences in MAZ-Ab titers were evident between ACS subtypes (*p* = 0.682). No associations between MAZ-Ab OD and conventional CVD risk factors were identified. Large-scale testing revealed no prognostic stratification regarding reinfarction (OR 1.04 [95% CI: 0.94–1.16]; *p* = 0.436).

**Conclusion:**

MAZ-Ab OD was higher or all ACS phenotypes compared with controls. Given current understanding of MAZ-Ab function, these findings support an autoimmune component to CVD independent of conventional risk factors and indeed the extent of end-organ damage.

## Introduction

Atherosclerosis is the pathophysiological process behind most cardiovascular, cerebrovascular, and peripheral vascular diseases. Consequently, it is associated with considerable morbidity and mortality (1). Numerous factors such as dyslipidemia, smoking, arterial hypertension, diabetes, and abdominal obesity appear to promote atherosclerotic plaque development within arterial vessel walls (2). Recently, the influence of multiple inflammatory and autoimmune promoters in atherosclerosis has been evaluated (3). While exact mechanisms remain unknown, autoantibodies such as anti-phospholipid antibodies, anti-oxidized low density lipid (oxLDL) antibodies, anti-phosphorylcholine antibodies, anti-apoA-1 IgG antibodies, and anti-heat shock protein antibodies have all demonstrated associations with atherosclerosis (3–5).

### MYC-Associated Zinc Finger Protein (MAZ)

MAZ is a synonym for serum amyloid A binding protein 1 (SAF-1), the function of which appears variable and incompletely understood. Activation and induction of MAZ by minimally modified LDL and lipopolysaccharides have been shown, leading to expression of serum amyloid A (SAA), a known inflammation-responsive protein within macrophages (6, 7). SAA has been long considered influential in atherosclerosis, with SAA mRNA having been identified in the endothelial and macrophage foam cells of atherosclerotic lesions of coronary and carotid arteries. Subsequently, MAZ has proven to be a transcription factor in active macrophages within atherosclerotic plaques triggering the formation of matrix metalloproteinases (MMPs) (8). Active macrophages are the hallmark of vulnerable plaques, with a high tendency to rupture. Originally identified in patients with coexisting CVD and autoimmune disease, MAZ-Ab has recently been evaluated in atherosclerosis using ^18^F-Fluorodeoxyglucose (^18^F-FDG) positron-emission and computer tomography (9). ^18^F-FDG uptake in vessel walls is known to correlate with macrophage activity in atherosclerotic plaques (10), with calculated plaque burden correlating with serum MAZ-Ab optical density (OD) (9).

Data regarding the further MAZ function have been emerging, with evidence supporting a role in certain cancers as regulator of oncogene transcription and angiogenesis (11–14). Furthermore, MAZ is known to regulate various inflammatory response genes (15), and associations to Alzheimer’s disease have been suggested in transcription factor analysis of 1,372-probe gene expression signatures (16).

### Study Aim

The aim of this study was to examine the clinical relevance of MAZ-Ab in patients with a confirmed cardiovascular event, compared with healthy controls. Furthermore, the relationship of MAZ-Ab to traditional cardiovascular risk factors and its prognostic value were evaluated.

## Materials and Methods

### Acute Coronary Syndrome (ACS) Cohort

To assess the risk of premature ischemic heart disease, unselected adult patients below the age of 65 years admitted to Hannover Medical School between August 2007 and July 2011, and subsequently diagnosed with ACS were included. All patients provided both verbal and written informed consent before participation in the study, with the ethics committee of Hanover Medical School approving the study (Ethics Number: 2614). All patients completed a questionnaire assessing cardiac risk profile and provided a serum sample, which was subsequently used for MAZ-Ab testing. The questionnaire assessed age, gender, past-history of hypertension, hypercholesterolemia, statin treatment, diabetes, tobacco exposure, as well as previous vascular events including myocardial infarction and stroke. All patients underwent routine ACS diagnostics including laboratory investigations, electrocardiogram (ECG), and coronary angiography. Laboratory tests included N-terminal propeptide brain natriuretic peptide (NT-proBNP), cardiac TroponinT (cTnT), growth-differentiation factor-15 (GDF-15), total cholesterol, and low-density lipoproteins (LDL). Serum samples were stored at −70°C.

Acute coronary syndrome patients were divided into three groups: patients with ST-elevation myocardial infarction (STEMI), with non-ST elevation myocardial infarction (NSTEMI) and with unstable angina pectoris (AP).

Patients with AP required at least 1 angiographically documented stenosis ≥70% in a major coronary artery. Based on ECG changes and cTnT, using a decision threshold of 0.03 µg/L, STEMI or NSTEMI was diagnosed.

### Control Group

Serum samples of an apparently healthy control group, which has previously been described in detail (17), underwent MAZ-Ab testing. Control patients received cardiac magnetic resonance imaging with dobutamine or adenosine stress, 12-lead ECGs, and physical examination without any pathological findings. In addition, all patients exhibited normal serum creatinine, aspartate aminotransferase, alanine aminotransferase, thyroid-stimulating hormone, hemoglobin concentrations, leukocyte, platelet counts, oral glucose tolerance test, and N-terminal pro-B-type natriuretic peptide (NT-proBNP) levels. None were in receipt of current medications and had no conventional cardiovascular risk factors. All were aged ≥18 years at inclusion and previously provided written informed consent.

### MAZ-Ab Testing

MAZ-Ab has been detected via protein array technique in patients with CVD as described in an earlier publication (9). A specially developed anti-MAZ-antibody ELISA kit (EUROIMMUN AG, Lübeck, Germany) based on our previously described ELISA protocol was used to test serum for MAZ-Ab (9).

MAZ-Ab optical densities were compared between ACS patients and controls, as well as within the ACS groups. Furthermore, MAZ-Ab values were correlated to traditional risk factors. To rule out an influence on MAZ-Ab by statins, LDL and cholesterol values of all patients with and without statins were analyzed additionally.

### Testing Prognostic Value of MAZ-Antibody after ACS

A well-defined group of 197 ACS patients, experiencing myocardial infarction, with up to 12 months subsequent follow-up were identified within the Platelet Inhibition and Patient Outcomes (PLATO) database (http://www.ClinicalTrials.gov NCT00391872) (18). An equal number (*n* = 199) of patients surviving 12 months follow-up without a subsequent spontaneous myocardial infarction were randomly drawn from the same cohort. Being matched for initial STEMI/NSTEMI, age, gender, and nationality the latter acted as controls. Serum taken at the time of initial admission was tested for MAZ-Ab in both groups.

### Statistical Analysis

Continuous variables were assumed non-parametric and tested using either the Mann–Whitney *U* test or Kruskal–Wallis test. Categorical variables were assessed using chi-square test or Fisher’s exact test. Correction for age and gender between groups was performed using Propensity Score matching, employing logistic regression with nearest neighbor matching and a 0.2 caliper. Prognostic relevance regarding reinfarction was calculated in conditional logistic regression. All *p*-values are two-tailed, with 95% cutoff being the declared level of significance. Statistical analysis was performed using IBM SPSS Statistics for Macintosh, Version 23 (IBM Corp. Armonk, USA), Prism 7 (GraphPad Software, La Jolla, CA, USA), and R Version 3.2.3 (R Foundation for Statistical Computing, Vienna, Austria).

## Results

In total, serum from 270 individuals was tested for MAZ-antibody. Of these, 174 (64%) were performed on patients at the time of admission for a cardiac event (ACS group). The remaining 96 (36%) were randomly selected healthy volunteers (Table 1). The median MAZ-Ab OD was significantly higher among ACS patients compared with controls (0.46 vs. 0.27; *p* = 0.001).

**Table 1:** Summary of the relevant laboratory parameters at the time of hospital admission for an acute coronary syndrome.

	AP (*n* = 14)	NSTEMI (*n* = 44)	STEMI (*n* = 116)	*p*
MAZ-Ab	0.55 [0.22–1.14]	0.52 [0.27–1.07]	0.37 [0.18–1.05]	0.682
TroponinT	19.1 [16.7–34.2]	92.4 [26.5–520.6]	163.9 [37.1–711.1]	0.002
ProBNP	203 [128–508]	267 [80–934]	123 [56–588]	0.156
GDF-15	1,450 [1,077–2,367]	1,399 [1,141–1,749]	1,503 [1,139–2,180]	0.505
CRP	2.7 [0.7–7.2]	2.9 [1.3–7.4]	2.3 [1.0–7.9]	0.655
Creatinine (μmol/L)	79 [70–110]	76 [69–89]	78 [68–90]	0.572
Cholesterol	220 [174–275]	197 [176–213]	182 [166–220]	0.210
LDL	150 [101–196]	139 [117–155]	131 [101–149]	0.604

*Values are grouped according to subsequently confirmed diagnosis. Other than an expected difference in Troponin, none of the other blood markers was associated with a particular diagnosis. In addition, none of these markers exhibited a quantitative correlation to MAZ-Ab titer*.

*MAZ-Ab, MAZ-antibody; AP, unstable angina pectoris; NSTEMI, non-ST elevation myocardial infarction; STEMI, ST-elevation myocardial infarction; GDF-15, growth-differentiation factor-15; LDL, low-density lipoproteins*.

### Influence of Age and Gender on MAZ-Antibody

**Figure 1:**
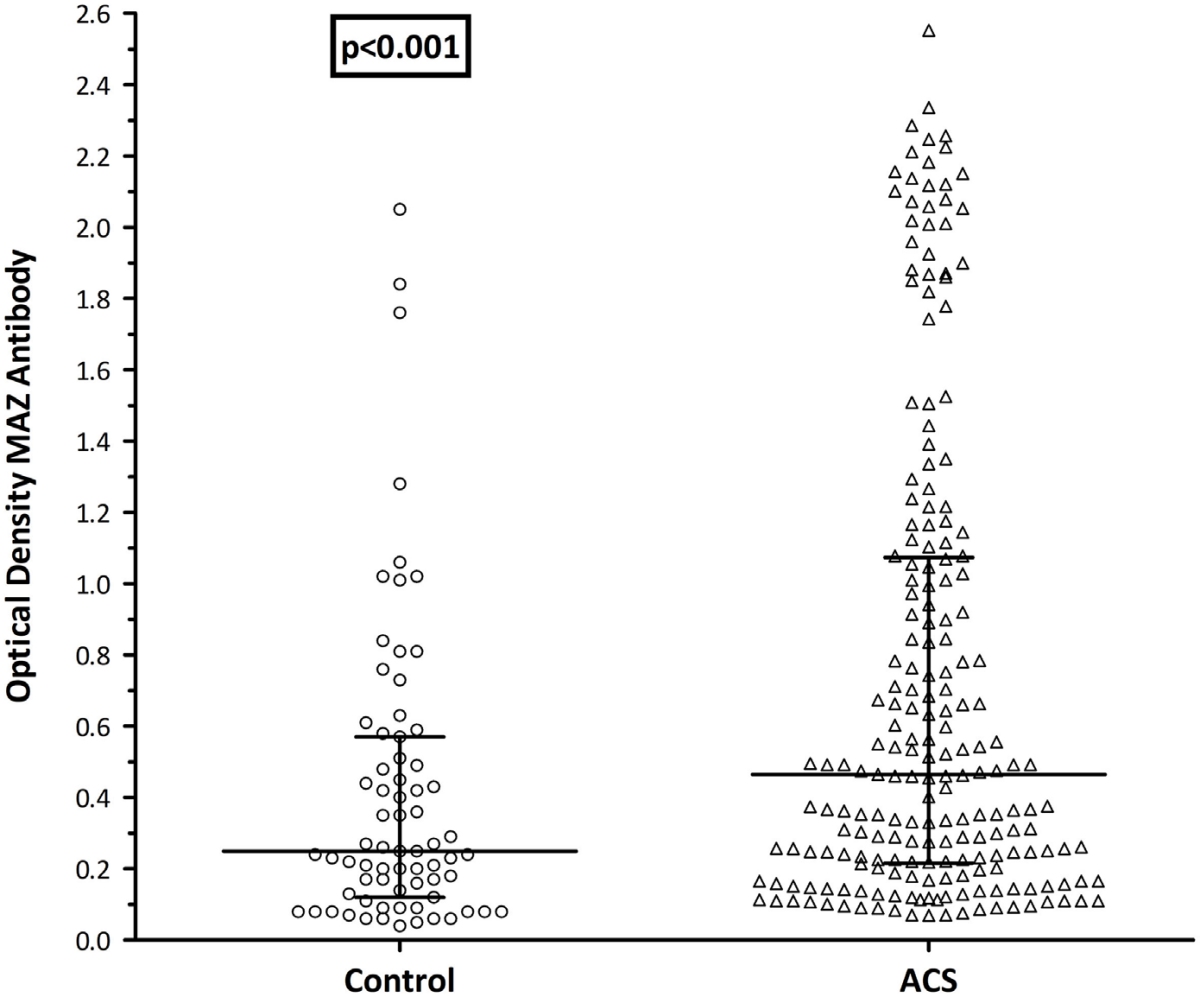
MAZ-antibody ± ACS: propensity score matched (age and gender), ACS group had significantly higher MAZ-Ab titers than the healthy control group. Control, control group; ACS, acute coronary syndrome patients; MAZ-Ab, MAZ-antibody.

Taking the entire cohort, the median age of the controls was significantly lower than the ACS group (41 vs. 53 years; *p* < 0.001). Similarly, a far greater proportion of the ACS group was male (87 vs. 47%; *p* = 0.001). To eliminate these potential confounders, a logistic regression analysis was performed using propensity score matching for age and gender between groups. In total, 206/270 patients (76%) were included in the sub-analysis, 168 from the ACS group and 38 control patients. The median ages were 49 [45–57] years among the controls and 53 [47–60] years in the ACS group (*p* = 0.46). While improved, a significant disparity in gender composition remained between groups, with a significantly greater male domination in the ACS group (83 vs. 68%; *p* = 0.003). Having eliminated age as a confounder, a significant difference in MAZ-Ab between groups remained with significantly higher ODs in the ACS group (0.47 vs. 0.24; *p* = 0.001 Figure 1).

### MAZ-Antibody Independent of ACS Phenotype

**Figure 2:**
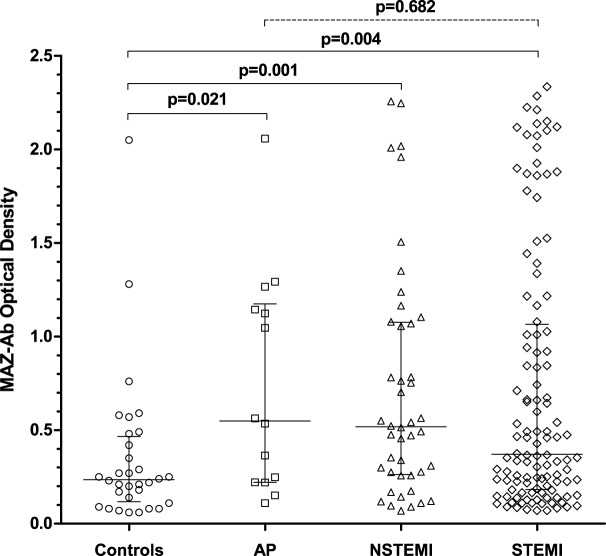
Comparing MAZ-optical density (OD) in the control group with the various acute coronary syndrome (ACS) phenotypes. MAZ-Ab OD was significantly lower in the control group compared with each ACS subgroup. Between ACS phenotypes, no significant differences in MAZ-Ab OD were observed, suggesting that MAZ-Ab was independent of the extent of myocardial damage occurring. Key: MAZ-Ab, MYC-associated zinc finger protein antibody; AP, unstable angina pectoris; NSTEMI, non-ST elevation myocardial infarction; STEMI, ST-elevation myocardial infarction.

Within the ACS group, 116/174 patients (67%) were diagnosed with a STEMI, 44 patients (25%) with NSTEMI, and 14 (8%) with AP. Within the different ACS phenotypes, no differences in MAZ-Ab OD were observed between groups (*p* = 0.682), all of which proved higher than that observed among controls (Figure 2), suggesting that MAZ-Ab was independent of the extent of myocardial damage occurring. Supplementing this further, a comparison of all the relevant blood markers (Table 1) revealed no significant correlations between these and MAZ-Ab OD.

### MAZ-Antibody and Traditional Cardiovascular Risk Factors

**Figure 3:**
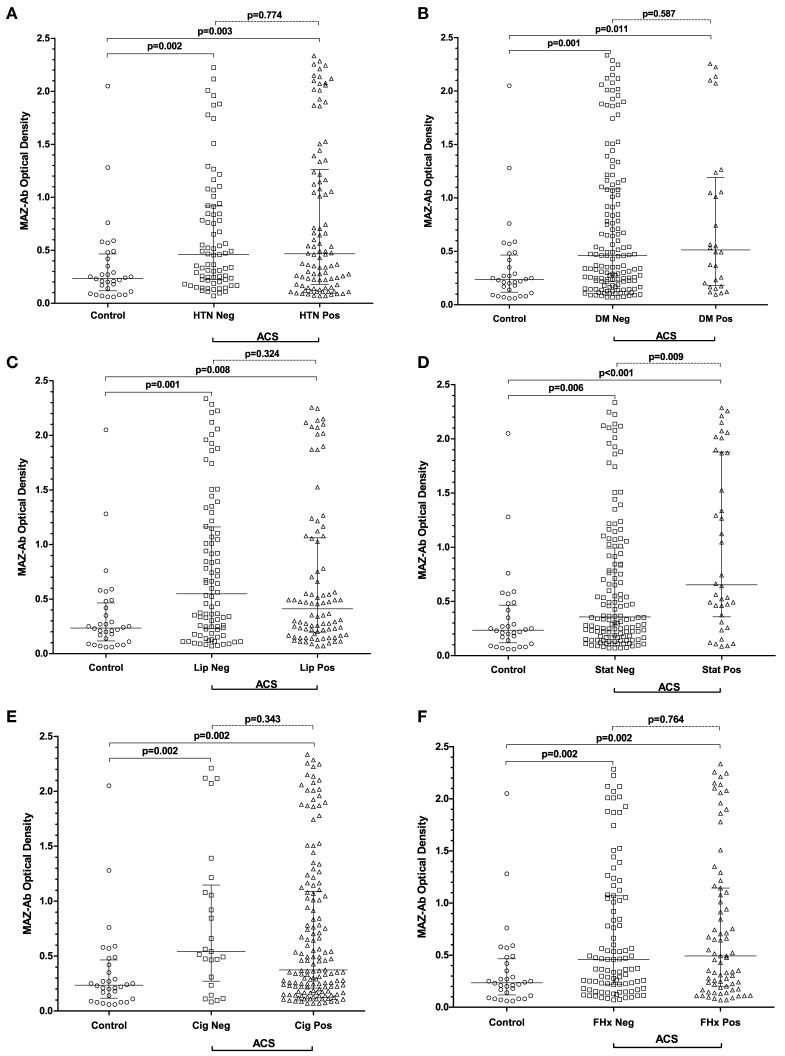
**(A–F)** Summarizing MAZ-Ab optical densities in all patients, with patients in the acute coronary syndrome (ACS) group being sub-classified on the presence or absence of specific cardiovascular risk factors. For all risk factors, MAZ-Ab optical densities were higher in ACS patients regardless of risk factor status compared with controls. Among ACS patients, only statin use resulted in a difference in MAZ-Ab optical density, with treated patients having higher values. Key: MAZ-Ab, MYC-associated zinc finger protein antibody; HTN, arterial hypertension; DM, diabetes mellitus; LIP, dyslipidemia; STAT, current treatment with HMG-CoA reductase inhibitors; Cig, any smoking history; FHX, any first-degree family history of cardiovascular disease.

Comprehensive data relating to the incidence of hypertension, diabetes mellitus, hyperlipidemia including treatment with statins as well as smoking habits and family history of CVD were available for all patients. No known risk factors were present among the entire control group. In the ACS group, the most prevalent risk factors were tobacco consumption (84%), hypertension (56%) and hyperlipidemia (49%). For all CVD risk factors assessed, MAZ-Ab optical densities were lower among control patients than in ACS patients without a given risk factor (Figures 3A–F). With the exceptions of evident hyperlipidemia at original admission and preexisting HMG-CoA reductase inhibitor use, no differences in MAZ-Ab OD were evident within the ACS group. Taken collectively, these findings would strongly support that MAZ-Ab is independent of hypertension, diabetes, dyslipidemia, smoking, and family history in terms of generating cardiovascular risk.

### MAZ-Antibody Independent of Cholesterol Levels and Use of Statins

The findings regarding existing statin use are less clear (Figure 3D). In total, 35 patients in the ACS group were taking statins at admission. Considering the recommended European Society of Cardiology cutoff values for blood lipid concentrations (cholesterol <190 mg/dL, LDL <115 mg/dL), 6/35 (17%) still had elevated cholesterol at admission. 59/109 (54%) of those admitted without statin had an elevated cholesterol, showing no significant difference between the two groups (*p* = 0.384). Of those on statin 22/35 (63%) and 75/109 (69%) of those without had raised LDL levels at admission (*p* = 0.59).

Patients on statins did, however, have significantly higher MAZ-Ab ODs (*p* = 0.009). It is worth noting that while these patients were older 58 [52–60] vs. 52 [46–58] years (*p* = 0.004), no association between age and raised LDL (*p* = 0.145) or cholesterol (*p* = 0.553) was observed. Similarly, neither cholesterol >190 (*p* = 0.945) nor LDL >115 (*p* = 0.946) influenced MAZ-Ab OD. An alternative explanation, however, may exist that the findings are the result of a selection bias, in that only high MAZ-Ab patients on statins subsequently experienced an event. This, however, remains speculative and requires further evaluation in future studies.

### MAZ-Antibody Has No Prognostic Relevance Regarding Reinfarction

Of the 197 PLATO patients experiencing a second cardiovascular event within one year of the initial ACS event, 42 died. No significant difference in MAZ-Ab OD between these patients and their 1997 matched controls was observed (OR 1.04 [95% CI: 0.94–1.16]; *p* = 0.436).

## Discussion

Taking a proven ACS as unequivocal evidence of atherosclerosis, this study clearly demonstrates a difference in MAZ-Ab OD on ELISA between these patients and healthy controls. This provides the first confirmed clinical association between MAZ-Ab OD and clinically apparent atherosclerosis. This augments existing data, which demonstrated an association between MAZ-Ab and the cumulative burden of inflammatory atherosclerosis on ^18^F-FDG-PET/CT scanning (9).

Previous research has shown that MAZ production is promoted by inflammatory interleukins including IL-1 and IL-6, along with TNF-alpha and oxLDL (8), all of which are known to increase cardiovascular risk (3, 4). Although the exact role of the transcription factor MAZ remains unclear, it has been implicated in macrophage function within atherosclerotic plaques (8, 19). The principal functions appeared to include inducing MMPs involved in atherosclerotic plaque rupture. Other studies have suggested a wider MAZ effect, such as influencing angiogenesis and inducing VEGF expression. The latter appears integral in triple-negative breast cancer cells (13, 14) as well as regulation of inflammation-responsive genes (15).

It is important to reiterate that current data suggests that MAZ-Ab is a biomarker of atherosclerosis rather than a particular cardiovascular disease. This study highlights this, when the various ACSs included are considered. In essence, the current classification of ACSs reflects the extent of end-organ, myocardial, damage. The measured cTnT values confirm this, increasing sequentially from angina pectoris, non-ST elevation and STEMIs (Table 1). No perceptible difference in MAZ-Ab OD across these groups was evident. Furthermore, no correlation between all cTnT values and MAZ-Ab OD were observed (Spearman rank, *p* = 0.391). Further comparisons with other known biomarkers revealed no relationship to MAZ-Ab OD, again supporting the concept that MAZ-Ab is myocardium independent.

It is, however, noteworthy that MAZ-Ab OD was independent of GDF-15. GDF-15 is an accepted independent prognostic biomarker for NSTEMI and acute chest pain, with increasing levels impacting negatively on 1-year post-event survival (20, 21).

It is well recognized that traditional cardiovascular risk factors are inextricably linked to the development of atherosclerosis. Considering only the patients in the ACS group, MAZ-Ab OD was not influenced by hypertension, diabetes, lipid or smoking status. However, comparing ACS patients lacking a particular risk factor with healthy controls returned uniformly raised MAZ-Ab ODs among the former.

MAZ antibodies were associated with statin use. Sub-analysis within the current cohort failed to identify any clear explanation for our findings. The most likely explanation appears to be selection bias and the demographics for statin use. Furthermore, it is reasonable to assume that all of those treated were at some point either diagnosed with dyslipidemia of unknown duration or as secondary prevention following a previous cardiovascular event and that the actual duration of statin treatment is unknown. All of these characteristics are likely confounders of increased atherosclerosis risk.

Given the limited follow-up in the ACS group we collaborated with the PLATO trial group to evaluate the prognostic value of MAZ-Ab following a vascular event, both in terms of survival and treatment response. Among these 396 matched patients, actual MAZ-Ab OD demonstrated no prognostic relevance, again suggesting that. MAZ-Ab OD may be a better marker for atherosclerosis than myocardial damage. This complements current understanding of the pathophysiological role of MAZ, a known transcription factor in active macrophages within atherosclerotic plaques (8).

Given the single-center, retrospective nature of this study there are several incumbent limitations that require consideration. The number of patients included is comparatively small, and significant demographic differences were evident between the groups, limiting somewhat the validity of data analysis.

In conclusion, however, this study corroborates clinically the findings of previous imaging studies, demonstrating an association between MAZ-Ab OD and proven atherosclerotic disease in the form of ACSs. The exact autoimmune role of MAZ-Ab in atherosclerosis may be related to plaque macrophage activity but more research into the pathophysiological function of MAZ is needed. Most importantly, current evidence suggests that the association between MAZ-antibody and atherosclerosis is independent of established conventional risk factors of atherosclerotic disease.

## Author Contributions

DE: study design, data collection, analysis, and writing of the manuscript. CW: data collection, analysis, and reviewing the manuscript. NB: study design, data collection, and reviewing the manuscript. WS and CD: data collection and review of the manuscript (technical aspects only). RS: critical review of the manuscript. KG and LW: data collection, analysis, and review of the manuscript. TW: study design and critical review of the manuscript.

## Conflict of Interest Statement

DE, NB, and TW have recently patented the MAZ-antibody (PCT/EP2016/066588). WS and CD are employees of EUROIMMUN AG and shareholders in EUROIMMUN AG. Their role in the current project was, however, limited to developing the standardized ELISA test used and testing the PLATO cohort. They were in no way involved in designing the study, data collection, or analysis and provided only critical review of the manuscript related to their involvement. KG: institutional research grant from AstraZeneca. LW: institutional research grants, consultancy fees, lecture fees, and travel support from Bristol-Myers Squibb/Pfizer, AstraZeneca, GlaxoSmithKline, and Boehringer Ingelheim; institutional research grants from Merck & Co. and Roche Diagnostics; consultancy fees from Abbott; and holds a patent EP2047275B1 licensed to Roche Diagnostics, and a patent US8951742B2 licensed to Roche Diagnostics.
